# SIAP: an intelligent algorithm for multiple prescription pattern recognition based on weighted similarity distances

**DOI:** 10.1186/s12911-023-02141-3

**Published:** 2023-05-04

**Authors:** Yifei Wang, Julia Xu, Jie Zhang, Hong Xu, Yuzhong Sun, Yuan Miao, Tiancai Wen

**Affiliations:** 1 Wangjing Hospital, China Academy of Chinese Medical Sciences, Beijing, 100102 China; 2 The University of Melbourne, Melbourne, VIC 3010 Australia; 3 Institute of Information Engineering, Chinese Academy of Sciences, Beijing, 100085 China; 4 College of Engineering and Science, Victoria University, Melbourne, VIC 3000 Australia; 5 Institute of Computing Technology, Chinese Academy of Sciences, Beijing, 100080 China; 6 Data Center of Traditional Chinese Medicine, China Academy of Chinese Medical Sciences, Beijing, 100700 China

**Keywords:** Prescriptions, Drug combinations, Electronic health records, Traditional Chinese medicine, Identification model, Intelligent algorithm

## Abstract

**Background:**

Clinical practices have demonstrated that disease treatment can be very complex. Patients with chronic diseases often suffer from more than one disease. Complex diseases are often treated with a variety of drugs, including both primary and auxiliary treatments. This complexity and multidimensionality increase the difficulty of extracting knowledge from clinical data.

**Methods:**

In this study, we proposed a subgroup identification algorithm for complex prescriptions (SIAP). We applied the SIAP algorithm to identify the importance level of each drug in complex prescriptions. The algorithm quickly classified and determined valid prescription combinations for patients. The algorithm was validated through classification matching of classical prescriptions in traditional Chinese medicine. We collected 376 formulas and their compositions from a formulary to construct a database of standard prescriptions. We also collected 1438 herbal prescriptions from clinical data for automated prescription identification. The prescriptions were divided into training and test sets. Finally, the parameters of the two sub-algorithms of SIAP and SIAP-All, as well as those of the combination algorithm SIAP + All, were optimized on the training set. A comparison analysis was performed against the baseline intersection set rate (ISR) algorithm. The algorithm for this study was implemented with Python 3.6.

**Results:**

The SIAP-All and SIAP + All algorithms outperformed the benchmark ISR algorithm in terms of accuracy, recall, and F1 value. The F1 values were 0.7568 for SIAP-All and 0.7799 for SIAP + All, showing improvements of 8.73% and 11.04% over the existing ISR algorithm, respectively.

**Conclusion:**

We developed an algorithm, SIAP, to automatically match sub-prescriptions of complex drugs with corresponding standard or classic prescriptions. The matching algorithm weights the drugs in the prescription according to their importance level. The results of this study can help to classify and analyse the drug compositions of complex prescriptions.

## Background

In clinical practice, diagnosis and treatment are complex processes. Doctors often prescribe a series of drugs for patients with chronic diseases, which is particularly common in elderly patients [1–4]. In medicinal prescriptions, multiple drugs may be grouped, but indications and importance levels are not often recorded. In addition, variations in patient demographics, infection sites, pathogenic bacteria, individual differences, and doctors’ experience may affect the drug prescription composition. Thus, it is difficult to consistently assess the efficacy of drugs and monitor drug‒drug interactions. For example, consider a critically ill patient with multiple organ failures that is admitted to a hospital with a severe lung infection. The doctor prescribes *linezolid* as the primary drug (for the most painful symptoms or signs of the patient) to treat a Streptococcus pneumoniae infection [5–7]. The doctor also administers *norepinephrine* to increase the patient’s blood pressure and kidney perfusion [8]. *Erythropoietin* is also used to maintain haematopoiesis [9]. These are auxiliary medications. In addition, the doctor may also prescribe *Saccharomyces boulardii* as a probiotic to repair intestinal dysbiosis [10, 11]. The doctor may also use *proton pump inhibitors* to prevent new lung infections caused by reflux aspiration [12]. In addition, *metoprolol tartrate* may be used to help the heart [13]. These are once again first-level drugs. The drugs have different mechanisms and target various disease symptoms and complications to reach an ideal treatment outcome. As another example, consider an elderly patient with chronic diabetic kidney disease that needs a variety of medications to control blood glucose, blood pressure, and blood lipids and reduce proteinuria [14, 15]. In Western medicine, there are corresponding guidelines, expert consensus, and standard prescriptions in various localities for single diseases and syndromes. The treatment process of traditional Chinese Medicine (TCM) also has guidelines, consensus, and treatment protocols, including traditional prescriptions for certain diseases. However, in clinical practice, the choice of the treatment plan is often influenced by factors such as geographic conditions, the patient's physical condition, genetic history, comorbidities, and the patient's working and living environment [16]. The understanding of this knowledge varies among physicians, and patient cases often require variation from the guidelines.

Electronic health records are effective documents in doctors' diagnosis and treatment processes depending on the patient situation [17, 18]. Electronic records also contain considerable information and knowledge that can be used for reference in the treatment of clinically relevant diseases. However, there are difficulties in conducting research on real-world electronic medical record data. In data analyses and research based on electronic medical records, the dataset size can range from hundreds to tens of thousands of data points. Assuming that there are ten drugs in a complex prescription with three options for each drug, there are nearly 60,000 combinations without considering the dose. A few tens of thousands of data points are insufficient to support statistical analyses. As an example, if three sub-prescriptions could be matched through analyses, with each prescription containing three or four drugs, with a division between the primary and secondary medications, the corresponding combinations may be reduced to a few dozen, or at most, a few hundred. The sub-prescriptions in this study refer to combinations of medications recommended by guidelines, consensus, or clinical protocols with high clinical efficacy and reduced toxicity. An effective algorithm can be designed to classify and match complex prescriptions with appropriate sub-prescriptions, which would be of great significance in acquiring diagnosis and treatment knowledge and evaluating drug efficacy and interactions.

Previous studies on the classification of Western medicine prescriptions were mostly limited to single aspects. The goals of these studies were mostly to understand the correspondence between disease categories and prescribing patterns or to investigate shared prescribing patterns of diseases. For example, using a topic model, Sungrae Park et al. [3] investigated the disease-prescription patterns association model. Vallano A. et al. [7] calculated and compared drug selection by population type. At present, there is a lack of research on prescription classification methods based on clinical prescription data to obtain diagnosis and treatment knowledge. The same problem exists in the study of TCM, which involved extracting combinations of formulas from TCM records and referencing the corresponding treatment outcomes. The Institute of Information on Traditional Chinese Medicine of the China Academy of Chinese Medical Sciences [19] and Hanqing Zhao et al. [20] proposed the intersection set rate (ISR) algorithm to identify and classify classical prescriptions in TCM. However, the algorithm can identify only one formula based on "yes" and "no" conditions, does not consider the drug's weight in the prescription and lacks clinically based practical considerations. Thus, a prescription classification algorithm that can effectively classify and match complex prescriptions to their corresponding sub-prescriptions is still lacking.

In this study, clinical data were integrated to propose a subgroup identification algorithm for complex prescriptions (SIAP) based on weighted similarity distance calculations. The algorithm automatically understands, classifies and divides complex prescriptions from several medical information systems into small subsets through artificial intelligence or data algorithms and matches these prescriptions to the corresponding sub-prescriptions. The proposed algorithm provides a new and efficient method for rapidly acquiring clinical diagnosis and treatment knowledge. Moreover, the proposed algorithm can assist young physicians and scholars in more efficiently understanding and exploring experienced doctors' diagnoses and treatment strategies.

## Methodology

In this study, we proposed a subgroup identification algorithm for complex prescriptions (SIAP). The algorithm can automatically classify and divide a large number of complex prescriptions into small subsets, which are then matched to the corresponding sub-prescriptions. The algorithm improves the identification and acquisition of clinical medication knowledge. The proposed algorithm can classify and match Western medicines to corresponding sub-prescriptions, as well as classify and match Chinese herbs to corresponding formulas. We also compared the SIAP algorithm with the ISR algorithm to evaluate the performance of the SIAP algorithm. The ISR algorithm was chosen because there is little research on prescription classification algorithms, and the ISR algorithm is currently the only work that has attempted to identify matches with typical prescriptions. A related platform based on the ISR algorithm has also been applied in the field of TCM.

**Fig. 1:**
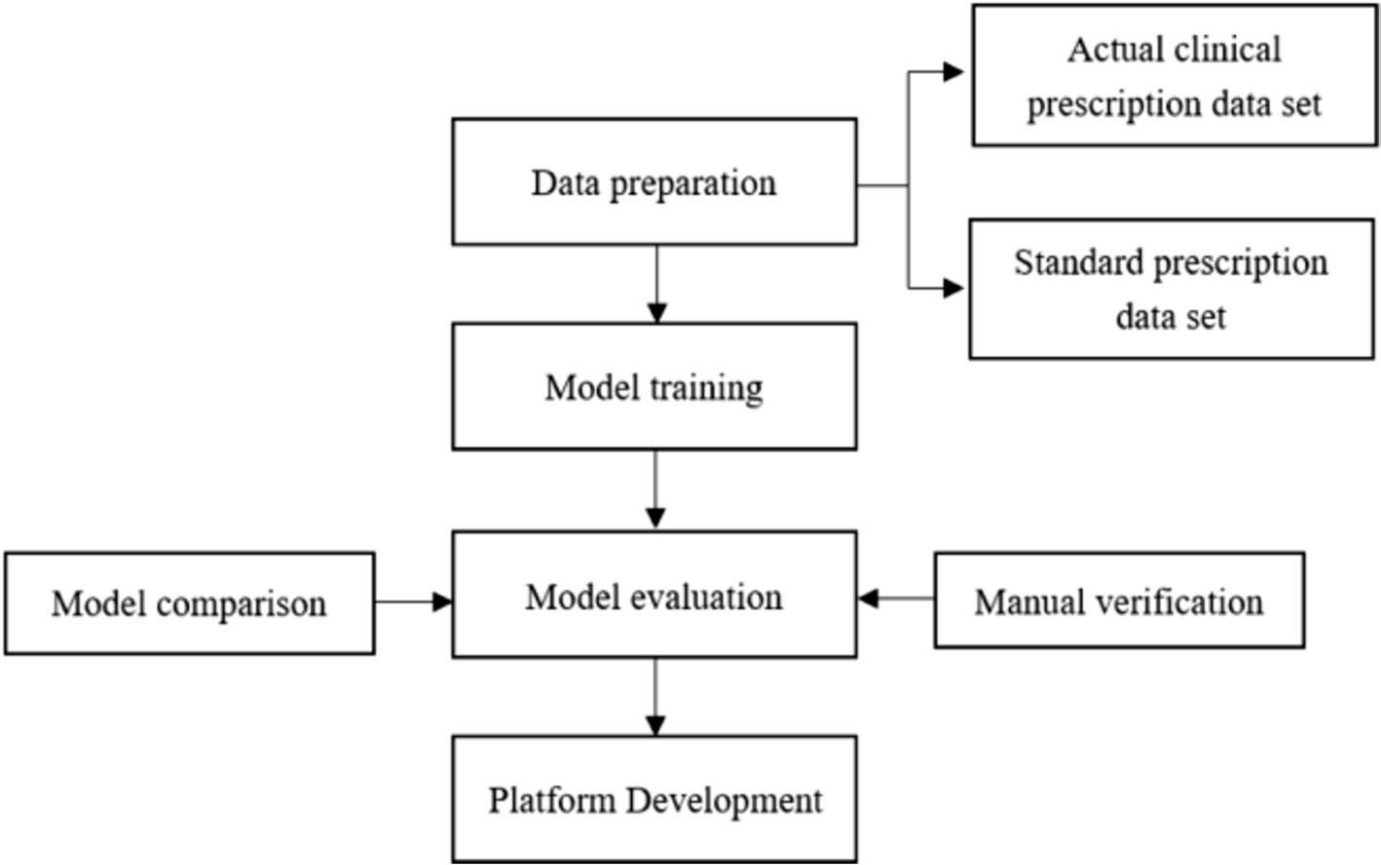
The overall framework of the study

The overall framework of this study is shown in Fig. 1.

- (1) A standard prescription database was built based on prescriptions composed according to guidelines, expert consensus, and expert experience. Real-world clinical cases from the archived data system of the Chinese Academy of Traditional Chinese Medicine Data Centre were extracted to build a collection of actual clinical prescriptions.
- (2) A logistic regression model was constructed based on the distance *d* between the samples to be identified and the corresponding subset of candidate prescriptions. Then, the relative importance weight *w* was calculated for each of the five classification pairs C1-C5 by prescription recognition. The similarity coefficient *sim* was calculated, and the recognition classification model was trained.
- (3) Based on the above information, a confusion matrix between the algorithm recognition results and manual marking results was built. The model training results were evaluated according to the accuracy, recall, and F1 value. A comparative analysis of the performance of the ISR algorithm with two SIAP sub-algorithms, SIAP-All and SIAP + All, was also carried out.
- (4) The model was validated based on a large amount of clinical data, and software for identifying TCM prescription treatment protocols was developed based on the SIAP + All algorithm.

### Algorithm design

The prescriptions from guidelines, expert consensus, and specialist experience were used to create a standard formulary, and each combination of drugs in the standard prescription was classified into four levels according to importance. Our examination of real-world datasets showed that the importance of each level varies in different domains. However, the first two levels are generally the most important, while the third and fourth levels are usually given less weight. The four-level classification method is applicable to most situations. The weight of each level can be obtained automatically by iterating through the corresponding real-world dataset. This process reflects the experience of various clinical practices and hospitals.

#### Algorithm framework

In the SIAP algorithm, different drugs are classified into five categories based on their weights. The weights in this study were defined according to the importance of the drug combination in the classical formulary or guidelines. C1, C2, C3, and C4 represent the four drug weight classes. A prescription including all drugs is defined as C5. C5 includes not only the C1-C4 drugs but also all drugs not included in C1-C4. This parameter is an optional parameter. On this basis, the distance coefficient *d* and weight coefficient *w* for C1-C5 were constructed. Finally, a comprehensive similarity evaluation index was constructed by weighted summation to evaluate the parameter *sim*, which is defined as the distance between the doctor's prescription and the standard prescription.

**Fig. 2:**
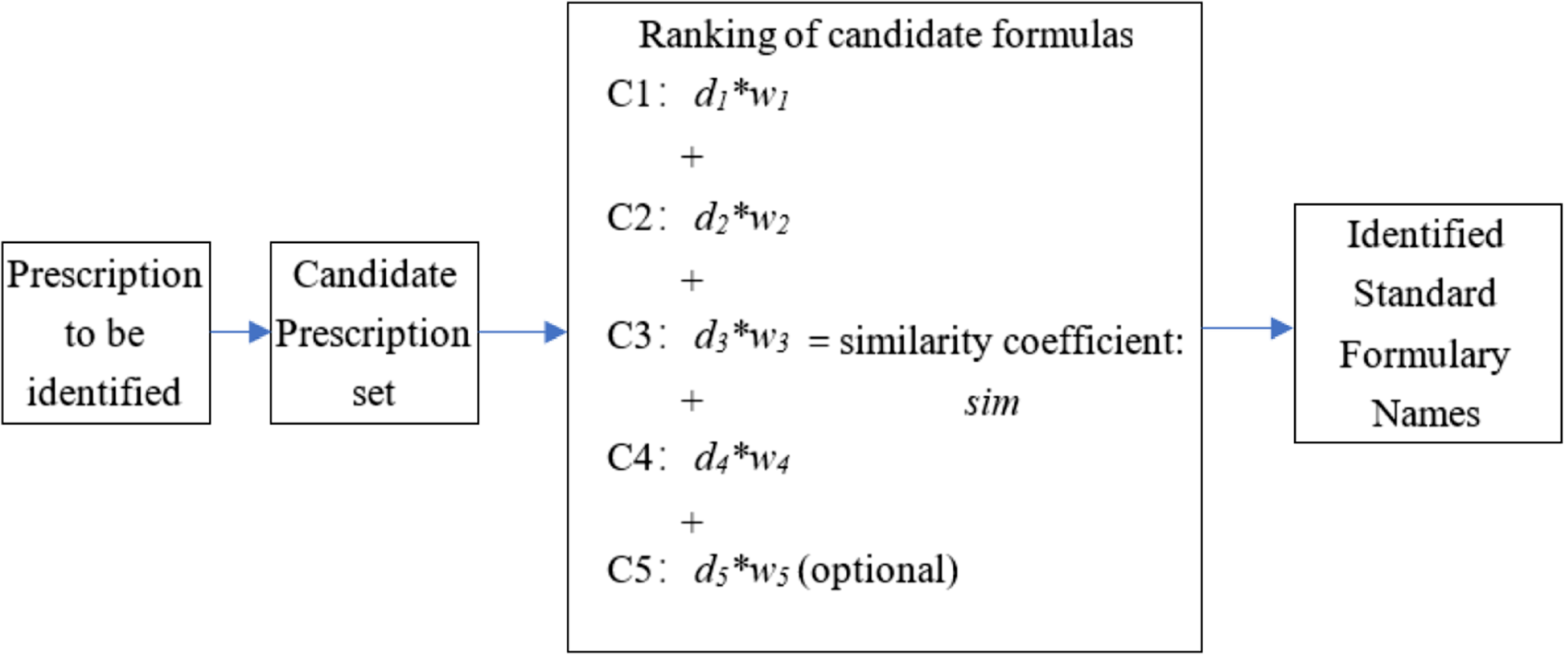
Algorithm framework

In this algorithm, for any prescription that contains multiple herbs, the algorithm first selects the candidate prescription set from the standard prescription set. The overall distance between the prescription to be identified and the standard compound is then calculated. The distance between C1-C5 is also calculated and multiplied by the corresponding weight and sum. Finally, the probability coefficients of all candidate formulas are obtained. The algorithm training process eventually leads to a suitable probability coefficient threshold, and the algorithm then outputs all standard prescriptions that are larger than this threshold (Fig. 2).

##### Generation of candidate prescription sets

Physicians may consider multiple therapeutic drugs when determining treatment prescriptions in clinical practice. Therefore, a simpler prescription pooling algorithm is used to select the candidate prescriptions. The algorithm is defined as follows:

1 $$C\;(A,\;B)\;=\vert A\cap B\vert$$

In the above equation, A represents the set of all drug compositions in the prescription to be identified, B represents the set of all drug compositions in a standard prescription, and C(A, B) represents the number of drug intersections present in the two sets. When C(A, B) ≥ 1, the standard prescription is included in the candidate prescription set.

##### Ranking of candidate prescriptions

###### Distance coefficient

In the prescription recognition algorithm, the distance coefficient is defined as the ratio of the intersection of the drug composition set of the prescription to be recognized and the candidate prescription drug composition set to the drug composition set of the candidate prescription. The distance coefficient is defined as follows:

2 $$d=D\left(A,B\right)=\frac{\left|A\cap B\right|}{\left|B\right|}$$

In the above equation, A represents the set of all drug compositions in the prescription to be identified, B represents the set of all drug compositions in a standard prescription, and *d* represents the proportion of the intersection of drugs present in the two sets to the set of medicines in the candidate prescription, so *d* ranges from 0 to 1.

The proposed algorithm includes five distance coefficients for different drugs in classes C1-C5. For D (A, B), A is the set consisting of all medicines in the prescription to be identified, and B represents the set of any class C1-C5 in the standard prescription. This formula can be applied to obtain the distance coefficients *d*_*1*_ ~ *d*_*5*_ for the five different sets.

###### Weight coefficients

The weight coefficients in this algorithm represent the relative importance of the five distance coefficients of C1-C5, labelled *w*_*1*_ ~ *w*_*5*_. The estimation was performed using the regression coefficient method, which was constructed as follows:

3 $$\log it(Y)=\beta0+\beta_1d_1+\beta_2d_2+\beta_3d_3+\beta_4d_4+\beta_5d_5$$

According to the definition of the logistic regression model, logit(Y) in the above equation is the natural logarithm of the quotient of the probability that is present prescription is judged correct while being incorrect, where* β*_*0*_ is a constant, and *β*_*1*_ ~ *β*_*5*_ are the coefficients of *d*_*1*_ ~ *d*_*5*_ in the regression model. To some extent, this model reflects the importance of independent variables to dependent variables. Variations in the independent variables *d*_*1*_ ~ *d*_*5*_ may affect the importance of the independent variables to the dependent variables; thus, it is necessary to standardize *β*_*1*_ ~ *β*_*5*_ to *β*_*1*_*'* ~ *β*_*5*_*'* [21]. The process is as follows:

4 $${{\beta }^{^{\prime}}}_{i}={|\beta }_{i}|\frac{\sqrt{3}{S}_{i}}{\pi }$$

5 $${w}_{i}=\frac{{{\beta }^{^{\prime}}}_{i}}{\sum {{\beta }^{^{\prime}}}_{i}}$$

where *β*_*i*_ represents the regression coefficient in the logistic model, *β*_*i*_*'* represents the standardized regression coefficient, and S_*i*_ represents the standard deviation of the independent variable *i*, with 1 ≤ *i* ≤ 5. The standardized regression coefficients *β*_*i*_*'* need to be normalized again to become the weight coefficients *w*_*i*_ used in this algorithm.

###### Similarity coefficients

To better assess the prescription identification results, the proposed algorithm uses a similarity coefficient to express the similarity between the prescription to be identified and the candidate prescriptions, which is calculated as follows.

6 $$\mathrm{sim}=\sum {d}_{i}{w}_{i}$$

where *sim* represents the similarity between the prescription to be identified and the standard candidate prescription, with *sim* ∈ [0,1]. *d*_*i*_ and *w*_*i*_ represent the *i-th* distance and weight coefficients, respectively.

### Experimental design

In TCM, prescriptions are often complex and composed of multiple herbs and formulas. The components are not often marked in clinical medical records, and the importance of the drugs is not delineated. This study used the treatment of diseases by herbal prescriptions as a dataset to evaluate the effectiveness of the proposed algorithm. In this experiment, 2000 herbal prescriptions from the data warehouse of the TCM Data Center of the China Academy of Chinese Medical Sciences were randomly selected to validate the SIAP algorithm.

#### Data collection and analysis

The data collation in this study involved constructing both a standard dataset and a clinical prescription dataset. The data were first deduplicated and standardized to remove cases with different names for the same medicine, e.g., "Xianlingpi" and "Epimedium" were standardized as "Epimedium". This study involves only prescribed medication and does not consider information about the medicine dose or frequency.

#### Standard prescription dataset

**Fig. 3:**
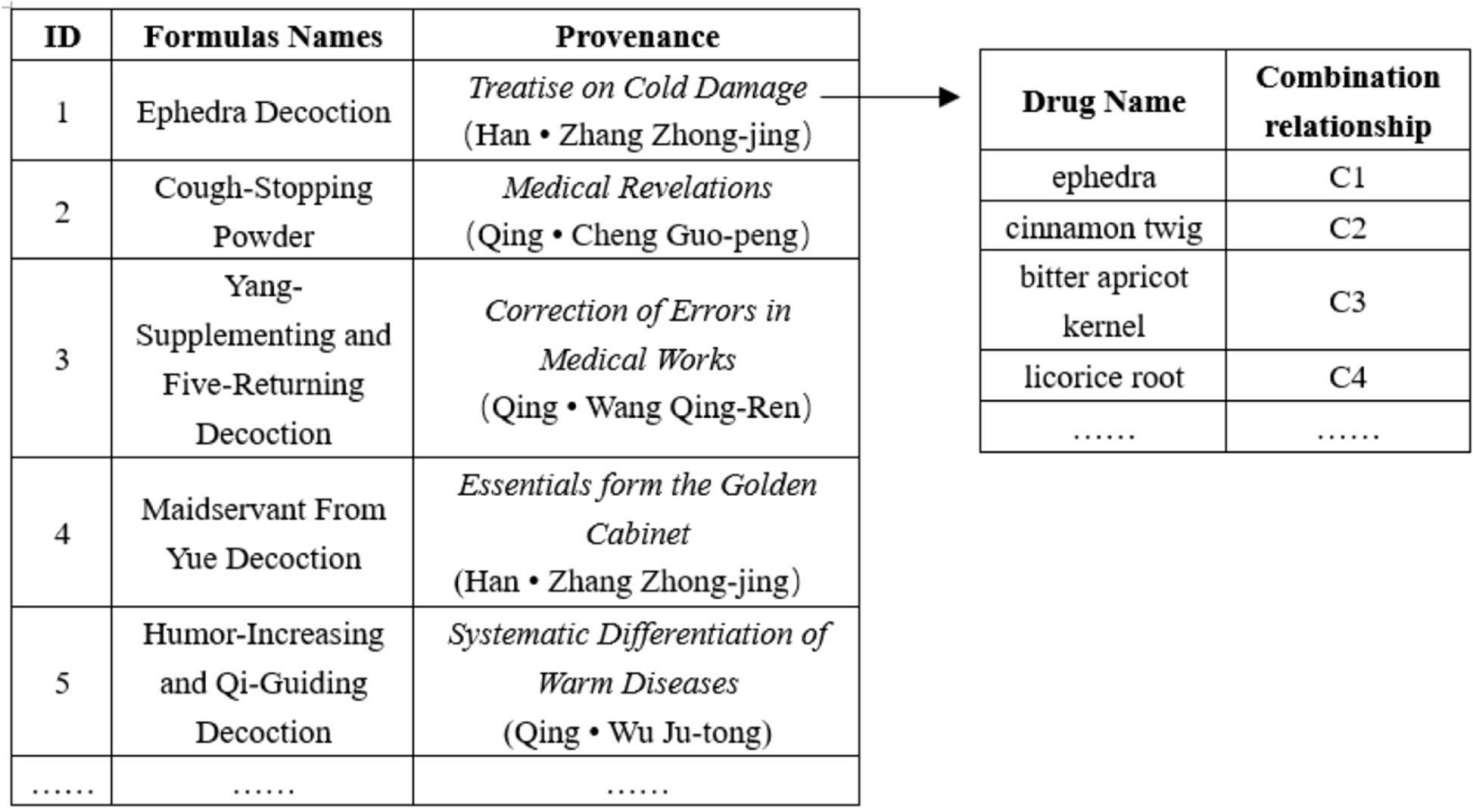
Schematic diagram of standard formula data set

The data of 376 formulas containing the information of "chief, deputy, assistant and envoy" (4 levels) were extracted from the "*Chinese medical formulas*" (10th edition) [22] and the *Countryside Langzhong* website [23] by Python 3.6.2. The name, origin, composition, and combined herb information of each formula were extracted, and the attributes of each medicine were labelled C1-C4 (Fig. 3).

#### Actual clinical prescription dataset

**Fig. 4:**
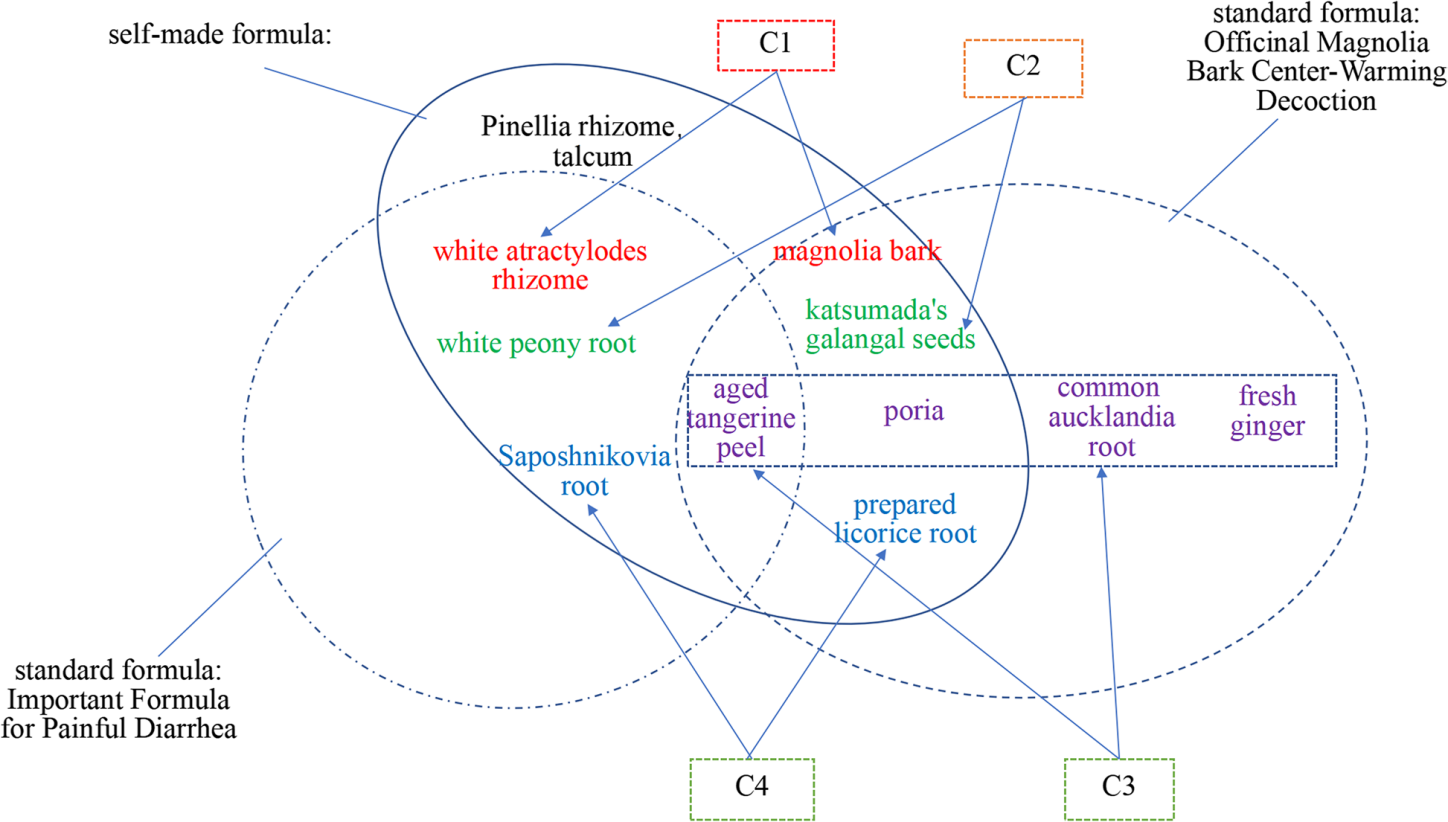
Relationship between actual TCM prescriptions and standard formulas

The Chinese medicine prescription data of real-world clinical cases were extracted from the data warehouse of the TCM Data Center of the China Academy of Chinese Medical Sciences. A total of 2000 herbal prescriptions were randomly selected, and 1438 prescriptions remained after duplicate prescriptions were removed. Two experienced TCM doctors manually identified the 1438 cases according to the standard formula dataset and marked the names of the standard formulas. If the prescription involved multiple standard formulas, the prescription was marked with multiple names. For example, if a prescription consisting of 10 herbs was determined to be mainly composed of Important Formula for Painful Diarrhoea (Tong Xie Yao Fang) and Officinal Magnolia Bark Center-Warming Decoction (Hou Po Wen Zhong Decoction), the prescription may fall under several groupings. In the Important Formula for Painful Diarrhoea, white atractylodes rhizome (Bai Zhu) belongs to C1, peony (Bai Shao) belongs to C2, aged tangerine peel (Chen Pi) belongs to C3, and saposhnikovia root (Fang Feng) belongs to C4. In the Officinal Magnolia Bark Center-Warming Decoction, magnolia bark (Hou Po) belongs to C1, katsumada's galangal seeds (Cao Dou Kou) belong to C2, aged tangerine peel (Chen Pi), poria (Fu Ling), common aucklandia root (Mu Xiang) and fresh ginger (Sheng Jiang) belong to C3, and prepared licorice root (Zhi Gan Cao) belongs to C4. Aged tangerine peel is classified as C3 in both the Important Formula for Painful Diarrhoea and the Officinal Magnolia Bark Center-Warming Decoction. Although the constituent herbs of common aucklandia root and fresh ginger in the standard formula of the Officinal Magnolia Bark Center-Warming Decoction did not appear in the present formula, the doctors determined that the prescription also contained Officinal Magnolia Bark Center-Warming Decoction. The reason is that other Chinese herbs in the prescription, such as the C1 and C2 drugs, all appeared in the prescription (Fig. 4). We randomly selected 70% (*n* = 1000) of the 1438 data points as the training set and the remaining 30% (*n* = 438) as the test set.

#### Training process

##### Calculation of the weight coefficients

According to Eq. (3), the weight construction process requires logistic regression with positive and negative sample data. Considering prescription recognition effects, in the final trained model, the negative samples may share some similarities with the positive samples. Therefore, this study used the intersection set rate (ISR) algorithm proposed by the Institute of Information on Traditional Chinese Medicine of the China Academy of Chinese Medical Sciences and Hanqing Zhao et al. to identify the prescriptions according to the training set data to increase the robustness of the model. We used the predicted data with incorrectly identified standard prescription names but matching probabilities greater than 0.5 (based on Eq. (2)) as negative examples in the training set.

Based on the above ideas, in this study, Eq. (1) was used to construct the set of candidate prescriptions corresponding to each sample in the training set. The distance *d*_*i*_ between all samples to be identified and the corresponding candidate formulas was calculated based on Eq. (2). Then, the logistic regression model was constructed using Eq. (3), and the relative importance weights *w*_*i*_ of the five distance coefficients C1-C5 were calculated using Eq. (4) and Eq. (5).

##### Similarity threshold training

According to Eq. (6), a similarity coefficient *sim* can be calculated for the formulas to be identified and the candidate formulas. The prescription to be identified may have a high similarity coefficient with several candidate formulas, which indicates that the formula to be identified may be a combination of several standard formulas. In this case, an appropriate threshold must be determined to specify the final formula.

In this study, the intersection set rate (ISR) algorithm was used as the basic algorithm, and the SIAP algorithm without all-set weights (SIAP-All) and the SIAP algorithm with all-set weights (SIAP + All) were compared with the basic algorithm. According to the principle of the ISR algorithm, Eq. (2) was directly used as the similarity coefficient, i.e., sim_ISR_ = *d*. In the SIAP-All and SIAP + All algorithms, the similarity coefficient *sim* was calculated based on Eqs. (3,4,5,6), where the SIAP-All algorithm did not include *w*_*5*_, while the SIAP + All algorithm included *w*_5_.

Instead of identifying the optimal similarity coefficient, in this study, the threshold was sequentially set to a real number between [0, 1]. The precision, recall, and F1 scores were calculated according to Eqs. (7,8,9), and the similarity coefficient *sim* that maximized the F1 score was selected. In addition, considering the possibility of confounding information or bias in the sample data, this study used the bootstrap (uniform sampling with replacement) method at different similarity *sim* thresholds. Two hundred samples were randomly selected from the training set during each iteration, and repeated sampling was conducted 100 times. The precision, recall, and F1 score were calculated every 200 samples, yielding a total of 100 precision, recall, and F1 scores. Finally, the model evaluation result of the similarity coefficient *sim* under this threshold had an average of 100 samples for the precision, recall, and F1 scores.

##### Evaluation indicators

According to the design of this study, the confusion matrix of the algorithm recognition results and the manual marking results was constructed (Table 1). The confusion matrix is the most commonly used assessment method and is associated with several evaluation metrics, mainly for dichotomous problems. The confusion matrix is also more effective for sample imbalance problems than traditional statistical indicators.

**Table 1:** Confusion matrix

Manually marked formula names	Formula name recognized by the algorithm	
	Consistent with manual making	Inconsistent with manual marking
Recognized by the algorithm correctly	${f}_{11}=\sum\limits_{n=1}^{N}\left|A\cap B\right|$	${f}_{12}=\sum\limits_{n=1}^{N}\left|B-\left(A\cap B\right)\right|$
Not recognized by the algorithm	${f}_{21}=\sum\limits_{n=1}^{N}\left|A-\left(A\cap B\right)\right|$	-

Note: n represents the sample number, N represents the sample size, A is the set of manually marked formula names, and B is the set of algorithm-identified formula names

In this study, the following three evaluation indicators were established [24].

###### Precision

The precision is defined as the percentage of prescriptions that were correctly identified by the algorithm.

7 $$Precision=\frac{{f}_{11}}{{f}_{11}+{f}_{12}}$$

###### Recall

The recall is defined as the percentage of manually labelled prescriptions that were recognized by the algorithm.

8 $$Recall=\frac{{f}_{11}}{{f}_{11}+{f}_{21}}$$

###### F1 score

The F1 score is a comprehensive evaluation indicator that considers the precision and recall.

9 $$F1=\frac{2PR}{P+R}$$

## Experimental results

### Basic information of the included prescriptions

A total of 376 standard prescriptions involving 419 herbs were included in this study, with each prescription containing an average of 7–8 herbs. The clinical prescription dataset included in this study had a total of 1438 prescriptions involving 445 herbs, and each prescription contained an average of 1–2 classical formulas on average. (Table 2).

**Table 2:** Basic prescription information

	**Standard Prescription Dataset**	**Clinical Prescription Dataset**
**Number of formulas**	376	1438
**Number of Chinese herbs, M (P25, P75)**		
C1	1 (1, 2)	-
C2	2 (1, 3)	-
C3	2 (1, 4)	-
C4	0 (0, 1)	-
C5	7 (4, 9)	11 (13, 15)

### Model training results

In this study, the ISR algorithm was used for the training set (*n* = 1000), and the similarity threshold was set to 0.5. A total of 8638 records were obtained, including 1653 positive examples (19.80%) and 6716 negative examples (80.20%) (Table 3).

**Table 3:** Training results of the intersection set rate algorithm

Formula identification results	Frequency	Percentage (%)
Correct	1653	19.80
Error	6716	80.20
Total	8638	100.00

In this study, when the logistic model for positive examples was used with the dataset, all coefficients were found to be statistically significant (*p* value < 0.05). Although the *β* coefficient for the whole formula was the largest, it had the largest standard deviation (*S*_*1*_ = 0.4525) for the C1 value. Its standardized coefficient (*β'* = 1.1391) and weights (*w*_*SIAP-All*_ = 0.4404, *w*_*SIAP*+*All*_ = 0.3418) were the largest, followed by those of C2 (Table 4).

**Table 4:** Coefficients related to the SIAP algorithm

Collection of Chinese herbs	*β*	*p value*	*S*	*β’*	*w*	
					**SIAP-All**	**SIAP + All**
**C1**	4.5659	0.0000	0.4525	1.1391	0.4404	0.3418
**C2**	3.6636	0.0000	0.4086	0.8254	0.3191	0.2477
**C3**	2.8336	0.0000	0.3490	0.5452	0.2108	0.1636
**C4**	0.4336	0.0127	0.3220	0.0770	0.0298	0.0231
**C5**	8.7578	0.0000	0.1545	0.7458	-	0.2238

**Fig. 5:**
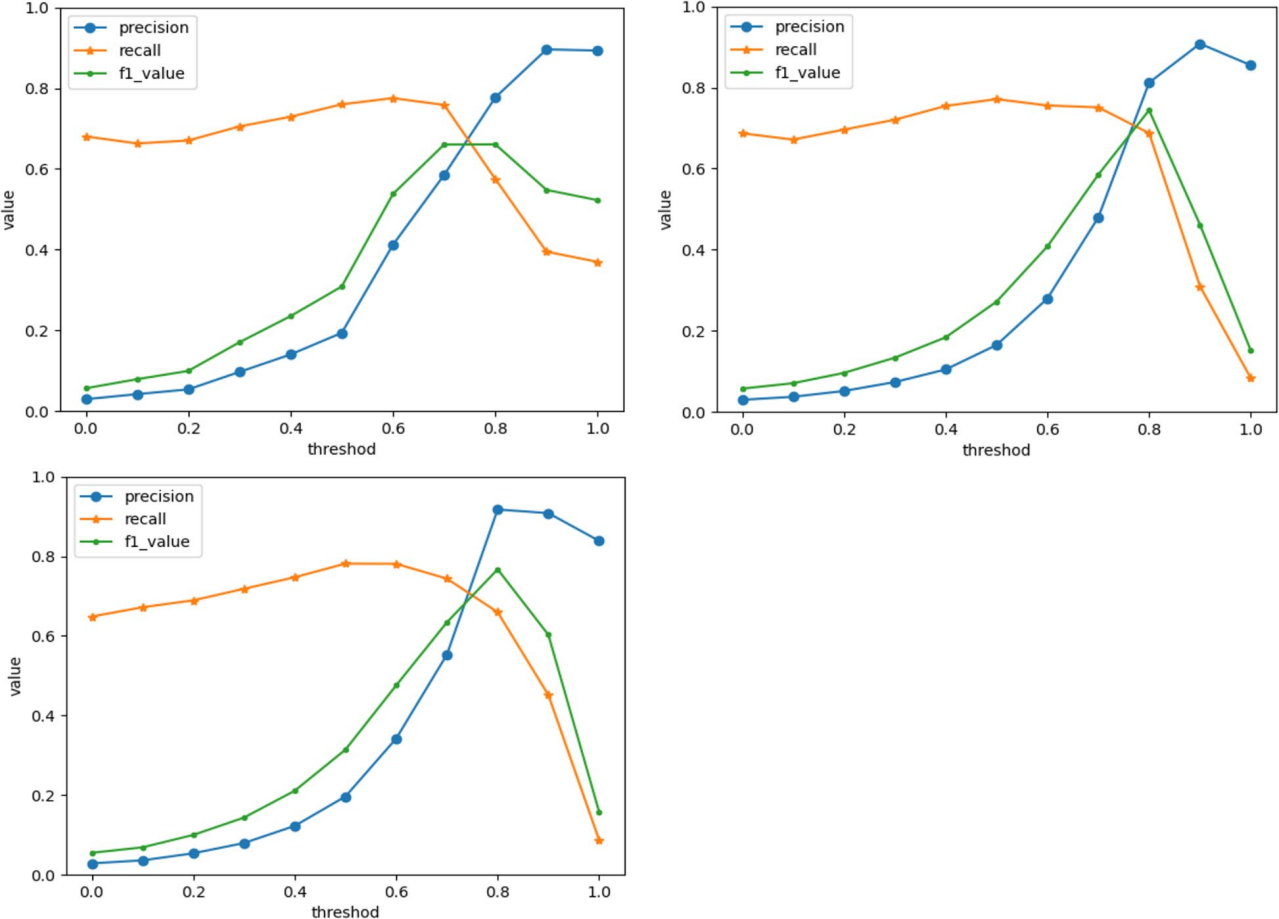
Training process of similarity threshold. **a**. ISR; **b**. SIAP-ALL; **c**. SIAP+ALL

After calculating the relative importance weights of the five distance coefficients of C1-C5 for formula identification, we determined the similarity thresholds. When the threshold of the ISR algorithm was set to 0.7, the F1 score was the highest. The SIAP-All and SIAP + All algorithms both had an optimal threshold of 0.8 (Fig. 5).

### Algorithm evaluation results

According to the above experimental design, three algorithms and standard datasets were used to identify the formulas of the prescriptions in the test set, which contained 438 labelled data points. Compared with the ISR algorithm, the F1 score of the SIAP-All and SIAP + All algorithms showed improvements of 8.73% and 11.04%, respectively, and the precision also increased (Table 5).

**Table 5:** Comparison of the three algorithms

Algorithm	Precision (P)	Recall (R)	F1 score (F1)
ISR: base	0.6022	0.7537	0.6695
SIAP-All	0.8420	0.6871	0.7568
SIAP + All	0.9430	0.6649	0.7799

### Manual validation of the prediction effects and platform development

**Fig. 6:**
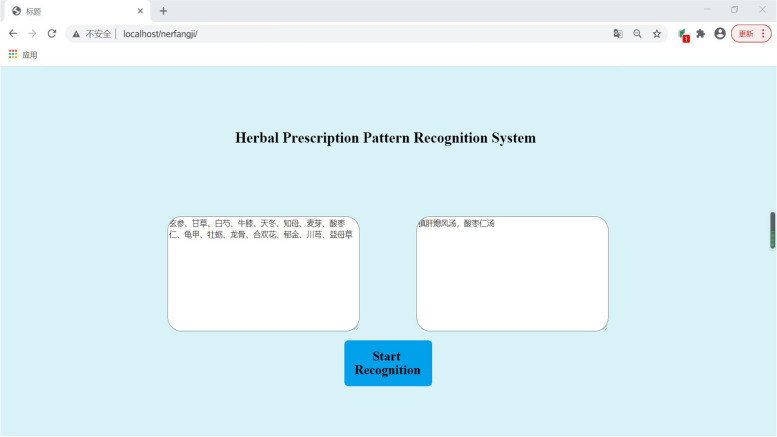
Example of TCM prescription recognition software

Based on the optimal SIAP + All algorithm for prescription identification and the results of 21,537 predicted samples, 5% of the prescription recognition results were sampled to manually verify the proposed algorithm. A total of 1077 data samples were randomly selected; out of the 1077 data samples, the algorithm correctly identified 875 samples and failed to identify 232 samples after a manual verification process by two TCM doctors. The algorithm correctly identified 81.2% of the samples in the dataset, and the results were as expected. On this basis, TCM prescription identification software was constructed based on the SIAP + All algorithm using Python web technology (Fig. 6). In the software, the user can directly input the composition of herbs to be identified into the identification box, and the system automatically outputs the name of the formula contained in the prescription.

## Discussion

In this study, the SIAP algorithm was successfully applied to the classification matching problem for multidrug prescriptions. The algorithm performed better than the baseline ISR algorithm in terms of the precision, recall, and F1 values. The model’s superior performance can be attributed to several aspects of the SIAP algorithm. First, in the model, the prescription classification is given a corresponding weight, which improves the accuracy of the classified sub-prescriptions and reduces the interference of invalid sub-prescriptions. A prescription is a medical document issued by a doctor to address a patient's condition that can be used as proof of the patient's medication, i.e., the patient's treatment plan [25]. In recent years, the extraction of therapeutic knowledge has been a significant issue in clinical practice. In the context of big data, prescription categorization and matching analyses can assist in the acquisition of therapeutic knowledge. Complex prescriptions often contain a variety of medications. The primary considerations in choosing a treatment plan and combination of treatment drugs are increasing efficiency while reducing toxicity. An appropriate drug combination can enhance the original effects of the drugs, correct their biases, and reduce their toxicities, thereby eliminating or reducing their adverse effects on the body [26–28]. Tuya et al. [29] studied the classification of prescriptions for heat syndrome in Mongolian medicine based on the fuzzy c-means (FCM) algorithm. They found that the FCM algorithm performed well, and the classification was relatively uniform across categories, in general agreement with the original literature. Clustering the results can assist in organizing and collating varied prescriptions, thereby improving accuracy and consistency. However, the algorithm considers the training categorization on a multidrug basis and does not consider the class weights; therefore, there is still a gap in its application in clinical practice. There has been relatively little research on prescription classification methods, and most previous research has focused on identifying prescription abuse and invalid prescriptions [30, 31]. This study introduced the concept of weighting to the traditional ISR algorithm and the similarly threshold. As a result, the new algorithm showed significant improvement in accuracy and applicability over the ISR algorithm in the classification and matching of sub-prescriptions.

The clinical performance of the SIAP algorithm constructed in this study was investigated using real-world clinical data and expert experience. The algorithm can quickly identify standard treatment schemes from a large number of treatment drugs. In addition, the algorithm can identify the treatment weights, as well as differentiate primary and secondary treatment drugs, by extracting the treatment schemes. In addition, the algorithm can collate medication recommendations for combined diseases and provide a reference for identifying combinations of Chinese and Western medical treatment protocols and combinations of physical therapies. The designed algorithm can efficiently extract the original prescription information from a large amount of clinical data, thus allowing the algorithm to comprehensively use clinical experience and rules of disease diagnosis and treatment. This development also has important guiding significance for junior doctors to improve their understanding of disease diagnosis and treatment. However, this study lacks the inclusion of underlying diseases, symptoms and signs, living areas, and other patient information; thus, the research results have certain limitations. In the future, further work will be done to expand the parameters of the included information and validation data to optimize the model.

## Data Availability

The data supporting the findings of this study are available from the corresponding author upon request. The code used in this research can be downloaded from the following GitHub repository: https://github.com/wtcsnake/SIAP.

## Abbreviations

SIAP: Subgroup identification algorithm for complex prescriptions
SIAP-All: C1-C4 weighting method without the all set weights
SIAP + All: C1-C5 weighting method
ISR: Intersection set rate
TCM: Traditional Chinese medicine
P: Precision
R: Recall
F1: F1 score
M: Median
(P25-P75): Interquartile ranges
